# A digital microfluidic system with 3D microstructures for single-cell culture

**DOI:** 10.1038/s41378-019-0109-7

**Published:** 2020-01-27

**Authors:** Jiao Zhai, Haoran Li, Ada Hang-Heng Wong, Cheng Dong, Shuhong Yi, Yanwei Jia, Pui-In Mak, Chu-Xia Deng, Rui P. Martins

**Affiliations:** 1 State-Key Laboratory of Analog and Mixed-Signal VLSI, Institute of Microelectronics, University of Macau, Macau SAR, China; 2 Faculty of Science and Technology-ECE, University of Macau, Macau SAR, China; 3 Cancer Center, Faculty of Health Sciences, University of Macau, Macau SAR, China; 4 Liver Transplantation Center, the Third Affiliated Hospital, Sun Yat-sen University, 510000 Guangzhou, China; 5 on leave from Instituto Superior Técnico, Universidade de Lisboa, Lisboa, Portugal

**Keywords:** Engineering, Electrical and electronic engineering

## Abstract

Despite the precise controllability of droplet samples in digital microfluidic (DMF) systems, their capability in isolating single cells for long-time culture is still limited: typically, only a few cells can be captured on an electrode. Although fabricating small-sized hydrophilic micropatches on an electrode aids single-cell capture, the actuation voltage for droplet transportation has to be significantly raised, resulting in a shorter lifetime for the DMF chip and a larger risk of damaging the cells. In this work, a DMF system with 3D microstructures engineered on-chip is proposed to form semi-closed micro-wells for efficient single-cell isolation and long-time culture. Our optimum results showed that approximately 20% of the micro-wells over a 30 × 30 array were occupied by isolated single cells. In addition, low-evaporation-temperature oil and surfactant aided the system in achieving a low droplet actuation voltage of 36V, which was 4 times lower than the typical 150 V, minimizing the potential damage to the cells in the droplets and to the DMF chip. To exemplify the technological advances, drug sensitivity tests were run in our DMF system to investigate the cell response of breast cancer cells (MDA-MB-231) and breast normal cells (MCF-10A) to a widely used chemotherapeutic drug, Cisplatin (Cis). The results on-chip were consistent with those screened in conventional 96-well plates. This novel, simple and robust single-cell trapping method has great potential in biological research at the single cell level.

## Microfluidics: Use of 3D microstructure enables single-cell culture

A novel microfluidic device enables researchers to isolate and culture single cells for use in drug testing and other experiments. To date, the high voltages required to capture cells on electrodes of a digital microfluidic (DMF) device have led to a reduced lifespan for the device and potentially damaged the cells. A team led by Yanwei Jia of the University of Macau overcame this by incorporating 3D microstructures into a DMF chip. This constrains the shape of cell culture droplets to isolate and capture single cells. With the use of low evaporation temperature oil and a surfactant, the system enabled them to use one quarter of the voltage of other designs for droplet transportation. The ability to isolate and culture individual cells will be of great value in addressing biological questions at the single-cell level.

## Introduction

Traditionally, cells are analyzed based on the responses of a large population cultured in Petri dishes or well plates^1,2^. However, in bulk analysis assays, the differences among individual cells (especially for primary tumor cells from patients) are masked, preventing us from obtaining a unique insight into the complex interaction between the environmental and single cellular activity. Single-cell culture and analysis remain in high demand for a full understanding of the cell-to-cell variability and for precision medicine.

Microfluidics has emerged as the most promising platform for single-cell analysis due to its characteristics in handling small volumes of samples. There are two main types of microfluidics: flow-based channel microfluidics and electric-based digital microfluidics (DMF). Single-cell culture has been investigated with channel microfluidics with one or no cells in each droplet for precise cell identification^3–6^. Microfluidic devices integrated with dielectrophoresis (DEP)^7,8^, optical tweezers^9–11^, or acoustic waves^12,13^ are powerful in trapping and manipulating single cells. Among the reported single-cell capture methods, microwell arrays fabricated in the flow channel have the highest single-cell capture efficiency. However, in all these studies, the cells were from the same inflow sample, where the stimuli had been already premixed. This setup greatly limited the number of drugs that could be screened on one chip. There is a possibility that droplets may diffuse away in some designs, with certain cells being lost. The problems arise from the characteristics of channel microfluidics, where droplets are generated and analyzed in a batch.

In contrast to channel microfluidics, digital microfluidics (DMF) is electric-based. This characteristic gives DMF advantages over channel microfluidics, such as individual droplet manipulation, multistep processes, flexible electric-automatic control and the potential for point-of-care. However, the size of a droplet on DMF (~0.3 μL) is much larger than can be achieved in channel microfluidics (1 nL), making it difficult to perform isolated single-cell analysis on a flat electrode. Wheeler’s group cultured cells^14–16^ by fabricating hydrophilic patches on electrodes for cell-based apoptosis assay applications^16^. However, multiple cells were captured in the droplet on the hydrophilic patch. To realize single-cell culture, Gidrol’s group demonstrated that by preparing a cell suspension with low concentration, single-cell isolation can be realized using DMF^17^. However, the single-cell capture efficiency was quite low, with one or two cells captured on an electrode. Lammertyn’s group reported that by fabricating many small-sized hydrophilic micropatches on an electrode, single cells can be captured for long-term culture. Nevertheless, the multiple hydrophilic patches greatly raised the actuation voltage needed to transport a droplet through this electrode^18^. As is well known, cells feel stress under certain electric field strengths and can even be lysed with a high electric field^19,20^. In addition, high-voltage actuation easily causes the dielectric layer to lose its insulating properties and break down, thus shortening the chip lifetime^21^. Therefore, the actuation voltage is desired to be as low as possible without compensating for the droplet movement efficiency, the cell viability and the observation of cells.

Physical and mechanical effects were also investigated for single-particle or single-cell trapping on DMF. For example, combining the function of gravity with the trapping geometry effect of negative dielectrophoresis, several research groups have realized single-particle or single-cell patterning on DMF^22–24^. However, all of these methods were used on a flat electrode surface. The most powerful 3D microstructures widely employed in channel microfluidics for single-cell trapping have been neglected in explorations of the DMF.

**Fig. 1:**
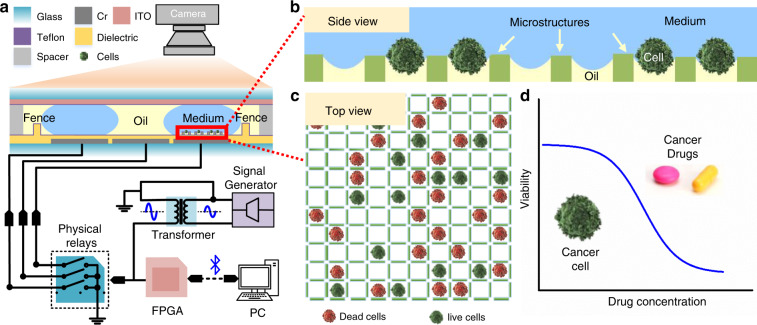
Schematic of the digital microfluidic (DMF) system for single-cell culture and drug toxicity tests. **a** Side view of the DMF chip and the electronic control system for droplet actuation. **b** Side view of the microstructures for interface formation and single-cell capturing. **c** Top view of the microstructure array for the drug toxicity test. **d** Example data obtained from an on-chip drug toxicity test based on the microstructure array

In this report, we present a DMF system (Fig. 1) for single-cell culture with innovative micropatterned arrays constructed by 3D microstructures fabricated on a DMF chip to trap single cells and to prevent the trapped cells from aggregating during a long-time cell culture. To minimize the influence of electric actuation voltage on cellular health, a low evaporation temperature and gas-soluble silicone oil with a solubility of oxygen several times greater than that in water^25–27^ and a fluorinated surfactant (F127) were introduced into the system to lower the actuation voltage to 36 V, which is 4 times lower than normally used (150 V). The oil quickly evaporated at 37 °C, the cell culture temperature, to expose the droplet to air for cell respiration. To demonstrate the technological advances, we ran the drug toxicity test by culturing breast cancer cells or normal cells with various concentrations of a clinically established chemotherapeutic reagent, Cisplatin (Cis), on-chip and compared with the off-chip scenario. The comparable results proved that the micropatterned arrays are effective for single-cell isolation and track monitoring during long-term culture. Due to the mature protocol of 3D microstructure fabrication on a DMF chip, the strong droplet control ability and the high single-cell trapping efficiency, the developed system has great potential for application in biological research at the single cell level.

## Results and discussion

### 3D microstructures for virtual channels, virtual chambers, and micro-wells

**Fig. 2:**
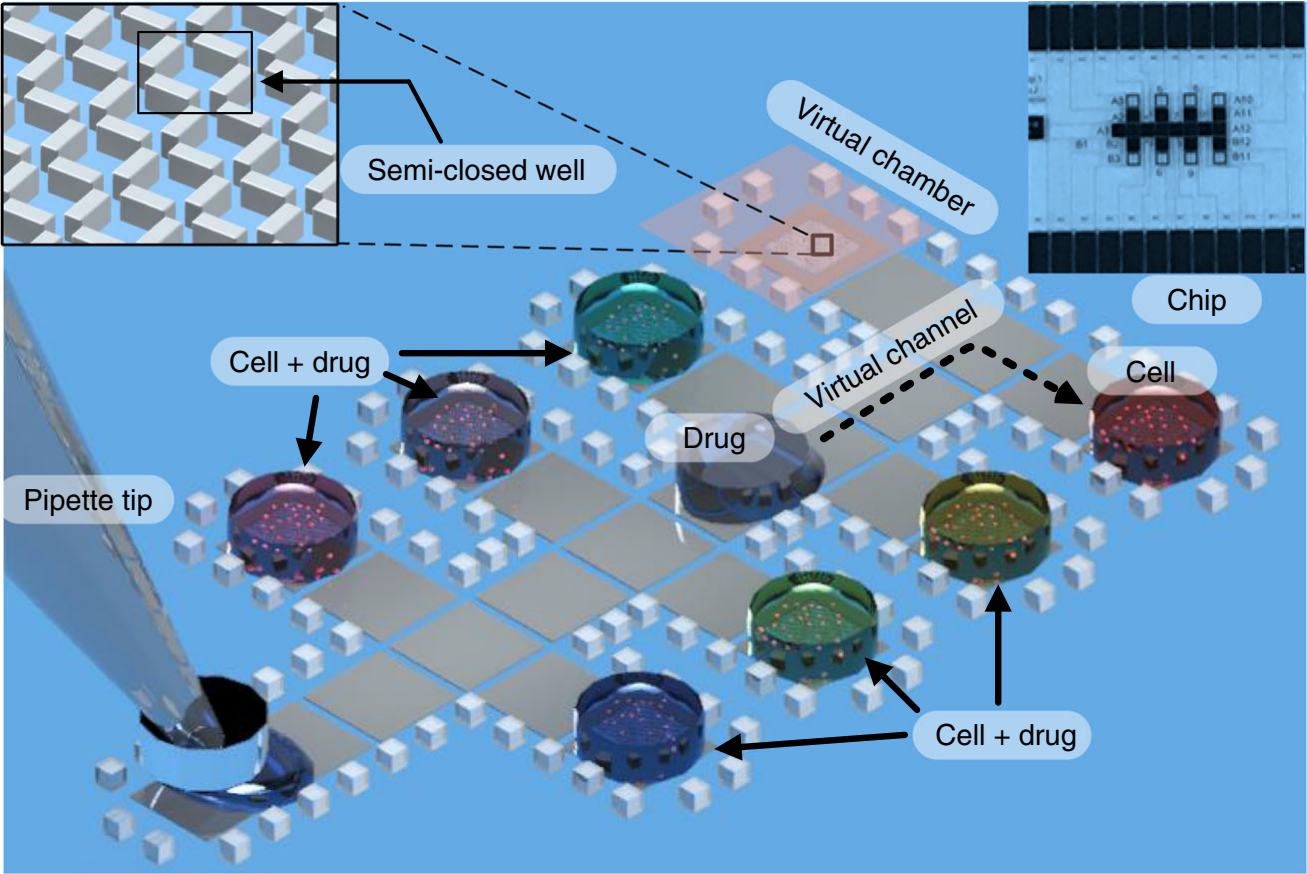
Schematic of the DMF chip with virtual channels and virtual chambers for the cell-culture-based drug sensitivity test. The inset images are the detailed microstructures and the whole picture of the DMF chip

For digital microfluidics (DMF), individual droplets are manipulated on an array of electrodes. In the sandwich-structured DMF chip, a chamber is formed by assembling a bottom plate with patterned electrodes and a top plate with a grounded conductive layer. This structure concentrates the electric field between the bottom and the top plates to lower the actuation voltage. When assembling the two plates together, there is an inevitable possibility that the space on one side is slightly thicker than the others. During a long-term cell culture on-chip, the pancake-shaped droplet may drift away from its original location to a deeper spot to lower its surface energy (ESI, video 1 and video 2). This would result in losing track of each droplet and the unexpected merger of two droplets. Wheeler’s group fabricated hydrophilic cell culture spots to avoid droplet drifting during cell culture. This required complicated chip fabrication, and later transportation of the droplet from the culture spot became a problem. In this work, we fabricated 3D microstructures as fences of 60 μm in height along the droplet transportation electrodes and the cell culture spots (Fig. 2). The distance between each fence post was approximately 300 μm, much less than the size of a droplet (1 mm). Surface tension prevented each droplet from getting through the fences, while the medium oil still freely moved around the fences. Virtual channels and virtual chambers were formed by structuring the fences on-chip to hold the droplets at certain places. During the long-term cell culture, the droplets were held in place for observation and analysis.

**Fig. 3:**
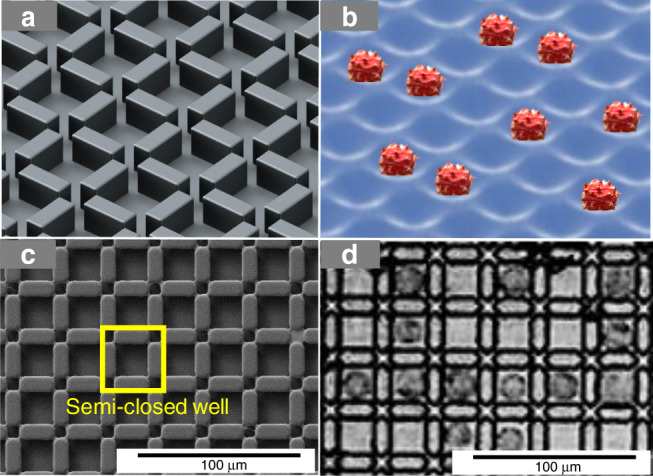
The scheme and image results of the microstructure arrays and the corresponding cell trapping results. **a** Microstructures designed on a digital microfluidic chip. **b** The interface formed between the microstructures and promoted single-cell formation. **c** The SEM results of the optimized microstructure arrays, arranged as wall arrays, for trapping single cells. **d** The corresponding results of trapping MDA-MB-231 cells under structures c after 24 h

In the sandwich-structured DMF chip, a droplet is normally in a pancake shape, with a plenary interface between the droplet and the substrates. Nevertheless, the shape of the interface is affected by environmental structures. When shallow 3D microstructures (Fig. 3a) existed on the plenary surface of the DMF chip, the restriction of the 3D microstructures beneath each droplet would force the droplet to form a curved interface due to the interfacial tension, as shown in Fig. 3b. We hypothesized that these curved surfaces would promote single-cell capture and storage.

**Fig. 4:**
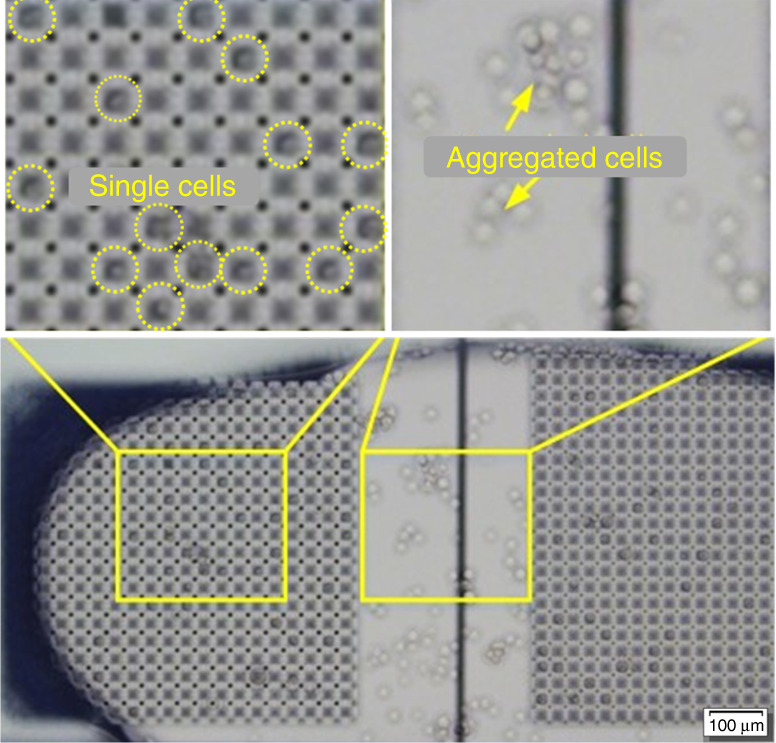
Optimized 3D microstructure array for single MDA-MB-231 cell culture on a DMF chip for 24 h

In this work, we tested various microstructure designs. Details can be found in the ESI, Fig. S1. The optimized structure is shown in Fig. 3a and Fig. 3c. The 3D microstructures were patterned as walls with a width of 10 μm, a length of 20 μm and a height of 10 μm. There was a small gap of 5 μm between the ends of each wall, forming a semi-closed well between the walls, as shown in the yellow frame in Fig. 3c. The single-cell trapping results are shown in Fig. 3d. As can be seen, cells were perfectly isolated from each other and stored in each semi-closed well. The long-term isolation of the single cells for observation and tracking is shown in Fig. 4. As shown, without the microstructure array, the cells tended to aggregate after 24 h, even when they were initially individually suspended in solution. However, in the presence of the microstructures, the single cells initially captured in the micro-wells remained in a single-cell state. This provided us an easy way to locate the single cells and keep track of the responses of a certain cell to various stimuli. The regular structure also made automatic data analysis possible for a final intelligent cell culture and screening system.

All the following experiments were based on the wall array design.

### Single-cell capture efficiency

**Fig. 5:**
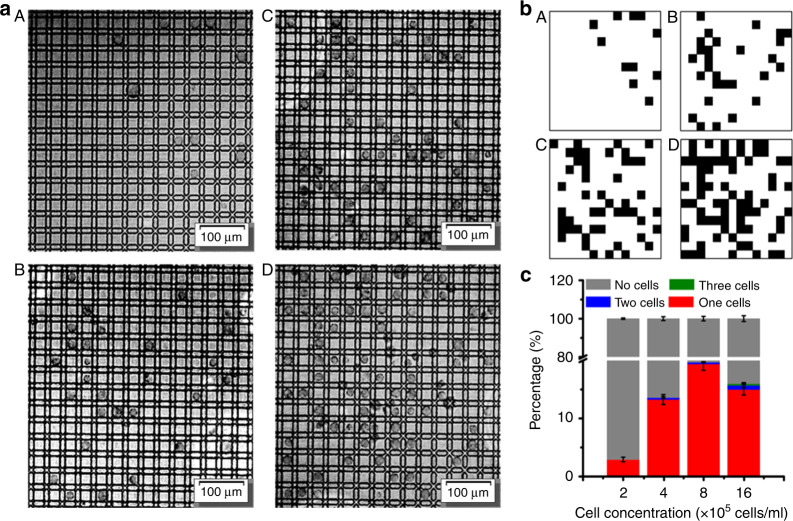
The image, schematic and statistical results of single cell trapping efficiency under the optimized microstructure conditions. **a** The image result. **b** The schematic result of a. The results of trapping MDA-MB-231 cells for A 2 × 10^5^ cells /mL, B 4 × 10^5^ cells/mL, C 8 × 10^5^ cells/mL, and D 16 × 10^5^ cells/mL, under the optimized 200 microstructure conditions. **c** The percentage of trapping MDA-MB-231 cells, classified as none, one, two and three cells under each cell concentration. The statistical results include 900 microstructures

Since the single-cell capture by the semi-closed wells shown above was passive, the capture efficiency was dependent on the cell concentration. Figure 5a shows images of the MDA-MB-231 cell distribution for cell concentrations of 2 × 10^5^, 4 × 10^5^, 8 × 10^5^, and 16 × 10^5^ cells/mL, respectively (panels A–D). For easy analysis, image analysis software (ImageJ) was used to determine whether each semi-closed well was occupied by cells or not, as shown in Fig. 5b. For the on-chip cell culture, each droplet contained approximately 200 cells. Note that in all cases, 100% of the input cells were loaded into the semi-closed wells as the whole droplet stayed on the patterned electrodes. Figure 5c shows the percentage of semi-closed wells occupied by single cells with various cell concentrations. As shown, the efficiency increased from 8% to 20% when the cell density increased from 2 × 10^5^ cells/mL to 8 × 10^5^ cells/mL. However, further increasing the cell density to 16 × 10^5^ cells/mL decreased the percentage of semi-closed wells occupied by single cells. Some of the semi-closed wells were occupied by two or more cells due to the high density of cells. In the following experiments, 8 × 10^5^ cells/mL was used as the optimized cell concentration for single-cell culturing.

### Oil film with surfactant for single-cell culture on chip

For cell culture on a DMF chip, droplets containing cells were open to the air for cell aspiration during culturing. However, in the absence of medium oil, the droplet transportation required a higher actuation voltage^28–31^. Although transportation only took a short time during the whole process, the strong electric field may still affect the response and growth of cells on-chip. Brassard et al. reported a water-oil core-shell structure able to lower the actuation voltage^32^. However, the construction of the core-shell structure required a precise water-oil ratio, making it difficult to realize in the cell culture system. In this work, we introduced a low evaporation temperature oil (PSF silicone oil, 1cSt) together with an inert fluorinated surfactant, F127, to help droplet transportation while maintaining cell aspiration during long-term cultures.

As shown in Table 1, for the normal electrodes, when the droplet was open to air, a 295 V actuation voltage (the highest voltage our system can provide) could hardly actuate the droplet under our sandwich-structured DMF setup. The existence of oil or surfactant alone lowered the actuation voltage to 202 V. The voltage in the absence of oil or surfactant in this work was slightly higher than reported^33–36^. This result may be due to the difference in system setups, such as the electric actuation frequency^32,33^ or thickness of the dielectric layer^35,36^. However, in the presence of both oil and surfactant, the voltage used for moving a droplet was 36 V, much lower than that normally used in the literature, that is, approximately 150 V. The significant difference may be caused by the change in the contact angle in the presence of surfactant. The microstructures on the electrodes increased the threshold of the actuation voltages under all conditions. The droplet was barely movable with a 295 V actuation voltage without the addition of oil and surfactant. However, with or without the microstructures, the addition of oil and surfactant significantly lowered the voltage by approximately 6-fold compared to the case of no oil/surfactant, from 295 V to 36 V or 50 V.

**Table 1:** The actuation voltage for the normal electrodes and patterned electrodes under different cell culture conditions on DMF chip

Conditions	Actuation voltage (V)	
	Normal electrodes	Patterned electrodes
No oil, no surfactant	295	295
Oil	202	295
Surfactant	202	295
Oil and surfactant	36	50

The effect of the spacing between the top and bottom electrodes on the actuation voltage was slightly more complex. Decreasing the spacing would increase the electric field, which would require a lower actuation voltage. At the same time, a narrow spacing would also increase the surface/volume ratio of each droplet, which would require a higher actuation voltage. The movement smoothness also depends on the viscosity of the droplet. Empirically, a droplet moves best when the electrode size-to-space ratio remains between 3 and 5. In our experiments in this work, the electrodes were 1 mm × 1 mm, and the spacer was 200 µm, yielding a ratio of 5.

The chip surface adsorption problem needs to be addressed before carrying out the on-chip applications. To quantitatively demonstrate the surface adsorption under different conditions, we ran a droplet containing 10 and 100 mg/mL recombinant eGFP (Recombinant Enhanced Green Fluorescent Protein, Beyotime, P7410) across the electrodes 10 times, 50 times or until the droplet stopped moving. Because the eGFP droplet could not be actuated at all under the air condition or oil conditions with a 295 V actuation voltage, only the conditions of the surfactant and oil and surfactant were measured. The fluorescence on an electrode was measured before and after transposing the eGFP droplet. Not much fluorescent difference was observed for either condition over the 50 actuations (Fig. S2), which suggested that the surface adsorption could be neglected for a certain number of actuations under the oil and surfactant conditions.

**Fig. 6:**
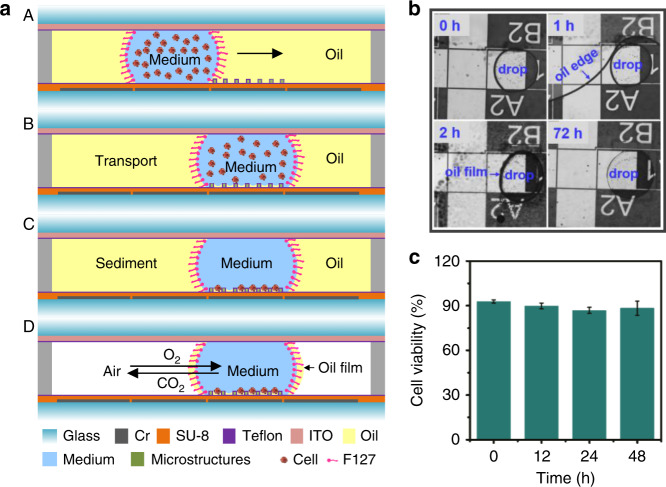
The scheme, cell suspension droplets state and cell viability results under the optimized cell culture conditions for a long time. **a** Parts **A**–**D** The scheme of moving the cell suspension to the virtual chamber, single-cell formation and the oil evaporation process. **b** Time dynamics research of the MDA-MB-231 cell suspension under the optimized cell culture conditions on a DMF chip for 0 h, 1 h, 2 h, and 72 h. **c** MDA-MB-231 cell viability results under the optimized cell culture conditions on a DMF chip

Figure 6a schematically illustrates the mechanism of single-cell culture in the presence of oil and surfactant. Once the droplet was transported to the cell culture chambers (A part of Fig. 6a), the chip was put into a humidified cell culture cabinet (37 °C, 5% CO_2_) for a long-time culture (B part of Fig. 6a). During the static stage of culturing, the suspended cells sedimented into the semi-closed wells as single cells (C part of Fig. 6a). At 37 °C, the silicone oil evaporated in 2 h given its low evaporation point, leaving a thin film of oil at the interface, as shown in D part of Fig. 6a. The size of the droplet remained the same (Fig. S3), even when the medium oil seemed to completely evaporate during the long-term culture (Fig. 6b and Fig. S4). This can be attributed to the presence of F127. The hydrophobic tale of the surfactant tended to hold a thin layer of oil film at the interface between the droplet and air. This thin film of oil allowed air to be exchanged while at the same time kept the water from evaporation. The existence of a thin film of oil did not affect the cell viability. As shown in Fig. 6c, a 90% cell viability was achieved after 48 h of culturing, similar to the initial cell viability just after loading on-chip.

In summary, the introduction of a low-temperature evaporation oil and surfactant has many advantages. The thin film of oil formed between the sample and the DMF chip reduced the sample adsorption and contamination during the loading process. The actuation voltage for droplet transportation was significantly lowered to 36 V, therefore reducing the risk of damage to the cells. In addition, the oil evaporated during the incubation to allow cell respiration while maintaining the size of the droplet to stabilize the drug concentration in the droplet. Therefore, the oil-filled configuration (with Pluronic F127 in the droplet) was used in the following drug sensitivity tests.

### Drug sensitivity test on-chip

As demonstrated above, the semi-closed wells formed by 3D microstructures on DMF had a high single-cell capture efficiency and could isolate single cells at a certain place for a long time for cell observation and tracking. A drug sensitivity test was used to validate the reliability of the DMF system. In this work, we used Cis (Cisplatin) as a drug model to test the drug sensitivity of MDA-MB-231 breast cancer cells and MCF-10A normal breast cells. An off-chip drug sensitivity test in a 96-well plate was also run in parallel as a comparison to verify the effectiveness of the on-chip case. The dead cells were stained with ethidium homodimer-1 (EthD-1), emitting red fluorescence.

**Fig. 7:**
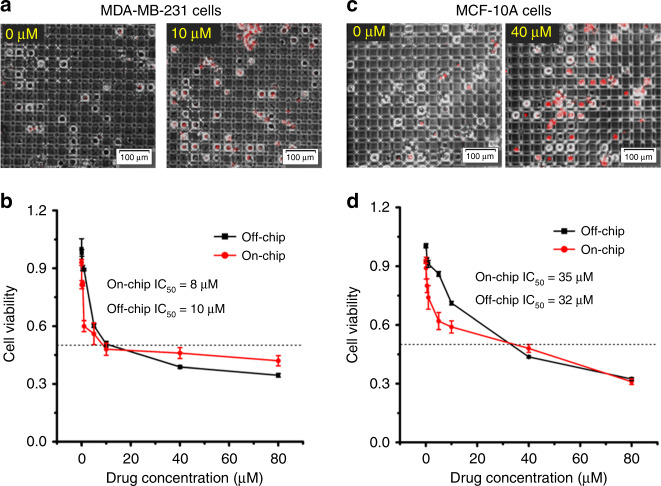
Cisplatin (Cis) was used as a model for the drug toxicity test on MDA-MB-231 breast cancer cells and MCF-10A normal breast cells. (**a** and **c**) The image results for on-chip assay results of **a** 0 μM, 10 μM Cis-treated MDA-MB-231 cells and **c** 0 μM, 40 μM Cis-treated MCF-10A cells for 24 h. (**b** and **d**) The drug toxicity test results for Cis-treated **b** MDA-MB-231 cells and **d** MCF-10A cells on our chip assay and off-chip (96-well plate)

Figure 7a, c show images of single-cell culture of the breast cancer cells and normal cells in the absence or presence of drug. As can be seen, for the control samples, both breast cancer cells and normal cells had good cell viability after 24 h of culture on-chip. In the presence of drug, more dead cells were observed for both cell lines. As single cells in semi-closed wells, the discrimination of dead cells from living cells was clear and easy to count, demonstrating the benefit of single-cell culture. Figure 7b, d show the viability of breast cancer cells and normal cells with Cis. As can be seen, the cell viability decreased when increasing the drug concentration either on-chip or off-chip for both cell lines. The IC_50_ value for the MDA-MB-231 breast cancer cells treated with Cis on-chip was 8 μM, comparable to the value tested off-chip, 10 μM (Fig. 7b). For the MCF-10A normal breast cells, the IC_50_ value for Cis on-chip was 35 μM, which was also comparable to that tested off-chip, 32 μM, (Fig. 7d). The slight difference between on-chip and off-chip was mainly caused by the total number of cells counted. Thousands of cells were counted off-chip, while only a few hundred cells were counted on-chip. All of the consistency validates the DMF system for drug screening on-chip with limited cell numbers.

## Conclusions

We set up a DMF system with 3D microstructures constructed on-chip for single-cell isolation and long-term culture. In the system, a low-evaporation-temperature oil and surfactant are innovatively employed to lower the actuation voltage to 36 V, 4 times lower than that normally used (150 V), for droplet transportation while retaining the open environment for cell respiration during long-term culturing. The result of the drug sensitivity test suggests that the designed structures are effective for single-cell trapping and evaluation for drug toxicity over time. Due to the mature protocol of 3D microstructure fabrication on a DMF chip, strong droplet controllability, and high single-cell trapping efficiency, the DMF system setup has great applications in biological research at the single cell level.

A potentially exciting application of our technology is precision medicine. If the cancer cells from biopsy samples can be properly labeled in the future, the true drug response of only the cancer cells can be monitored, and at the same time, those drugs toxic to normal cells can be monitored. All of these results will provide helpful information regarding the drug toxicity and side effects to doctors.

## Materials and methods

### System setup

The DMF system contained four parts: a DMF chip, an electronic control board, customer-written control software and a fluorescence microscope, as shown in Fig. 1a. An image of the real system setup can be found in the ESI, Fig. S5. The DMF chip was held by a 3D-printed chip holder and test clips, which connected the electric control to the chip via the exposed contact pads, with switches on the printed circuit board (PCB) for on-chip droplet actuation. A computer program^37^ was used to acquire the droplet position and execute droplet manipulation automatically by controlling the power switches. A signal generator was used to generate an AC actuation signal (0.5–10 Vrms, 2 kHz, sinusoidal wave), which was amplified to 30–300 Vrms by a transformer to charge the electrodes. The relationship between input voltage and output voltage after amplification can be seen in Fig. S6. The DMF chip was observed and imaged by a fluorescence microscope.

### DMF device fabrication

The DMF device consisted of three parts: the bottom plate, the spacer and the top plate. Arrays of on-chip electrodes (1 mm × 1 mm) were designed with AutoCAD and patterned on a glass substrate (31.5 mm × 31.5 mm) as the bottom plate. A layer of 10 μm SU-8 photoresist was first coated on the bottom plate as the dielectric layer, followed by a second patterned layer (60 μm thickness SU-8) as fences to prevent droplets from drifting^37,38^ and a third patterned layer (10 μm thickness SU-8) as a microstructure array to perform single-cell cultures. During the fabrication, a mask aligner (ABM, California, USA) was used for precise patterning of the dielectric layer, fences and microstructure array on the patterned chromium electrodes. After exposure, baking and development, a bottom plate with microstructure arrays on certain electrodes was obtained. The top plate was made of ITO glass (50 mm × 17 mm). 1.5 mm diameter holes for sample loading were drilled into the ITO glass using a laser cutting machine (ZKJ Laser, Shang Hai). Both the bottom and top plate were coated with Teflon (100 nm thickness) to promote smooth sample transportation. A 200 μm thick conductive adhesive tape was used as the spacer. The assembly illustration of the DMF chip can be found in the supporting information (Fig. S7).

### Reagents

IPA, acetone and ethanol were purchased from Millipore. The reagents used for photolithography, including SU-8 and SU-8 developer, were purchased from MicroChem. Amorphous Fluoroplastics Solution was purchased from the Chemours Company. Pluronic F127 was purchased from Sigma Aldrich (Oakville, ON, USA). Silicone oil (1 cSt) was purchased from Clearco, USA. MDA-MB-231 cells and MCF-10A cells were obtained from the American Type Culture Collection (Manassas, VA, USA). Dulbecco’s Modified Eagle’s Medium, fetal bovine serum (FBS), trypsin-EDTA and phosphate buffer solution (PBS) were purchased from Gibco. cis-Diammineplatinum(II) dichloride was purchased from Sigma. Ethidium Homodimer-1 (EthD-1) was purchased from Thermo Fisher Scientific.

### Cell culture

The MDA-MB-231 cells and MCF-10A cells were cultured in a humidified incubator (37 °C, 5% CO_2_). The growth medium for the MDA-MB-231 cells was Dulbecco’s Modified Eagle’s Medium (DMEM), supplemented with 10% (w/v) FBS, 2 mM L-glutamine, and 100 U/mL penicillin-streptomycin. The medium for MCF-10A was DMEM/F12, supplemented with 5% (w/v) Horse Serum, 20 ng/mL (w/v) EGF, 0.5 mg/mL (w/v) Hydrocortisone, 100 ng/mL (w/v) Cholera Toxin, 10 μg/mL (w/v) insulin and 100 U/mL penicillin-streptomycin. Both cell lines were cultured every 2–3 days for each passage at 2 × 10^5^ cells per cm^2^. Prior to the experiments, the cells were dissociated and resuspended in a fresh medium. The number of cells and cell viability were measured by cytometry and trypan blue exclusion.

### Cell viability assay under optimized cell culture conditions

We explored the cell viability assay for the oil-filled configuration (with 0.01% Pluronic F127 in the droplet) under the cell culture condition. 0.6 μl droplets (8 × 10^5^ MDA-MB-231 cells/mL) containing 0.01% Pluronic F127 and 2 μM EthD-1 were pipetted into the holes and dispensed by applying the actuation signal to the adjacent electrodes sequentially. When the droplets were moved to the virtual chambers, we placed the DMF chip in a humidified incubator (37 °C, 5% CO_2_). The experiments were performed in triplicate. Cells treated with a 60 °C water bath for 30 min and then with 0.01% Pluronic F127 and 2 μM EthD-1 were used as positive controls. After 0 h, 12 h, 24 h, 36 h, and 48 h, the DMF chip was observed for cell viability estimation under a fluorescent microscope.

### Drug sensitivity assay on DMF chip

One clinically established chemotherapeutic reagent, cisplatin (Cis), was used in the drug sensitivity test. MDA-MB-231 breast cancer cells and MCF-10A normal breast cells were used as the model cell lines. Briefly, the MDA-MB-231 cells and MCF-10A cells (8 × 10^5^ cells/mL) were aliquoted in 0.2 mL PCR tubes and then mixed with 0.01% Pluronic F127 and 2 μM EthD-1. After that, we filled the DMF chip with silicone oil (1 cSt). Then, cell suspensions and drugs in a series of concentrations were pipetted into the holes and then moved by applying an actuation signal to the adjacent electrodes sequentially towards the virtual chambers and mixed on the DMF chip. In our chip design and experiments, one path was for only one drug with concentrations from low to high, loaded onto the chip in a serial manner. Although some residues remained on the common path from the low concentration samples, they did not cause cross contamination and had little effect on the higher concentration samples. Then, the chips were placed in a cell culture dish containing wet paper towels and placed in a humidified incubator (37 °C, 5% CO_2_) for 24 h. Finally, we measured the red fluorescence of the cells via inverted fluorescent microscopy.

### Drug sensitivity assay off-chip

Determination of the half-effective concentration (IC50) of Cis for the MDA-MB-231 cells and MCF-10A cells was performed using a cell counting kit (CCK-8) assay^39,40^. Briefly, 1.0 × 10^4^ cells (the total volume was 100 μl) per well were seeded in a 96-well plate in the corresponding cell culture medium. They were then treated with various concentrations of Cis (with 0.1% (v/v) dimethyl sulfoxide (DMSO) treatment as a negative control and a cell culture medium without cells as a blank control) for 24 h. Then, 10 μL of CCK-8 solution was added to each well and incubated for 0.5 h. All experiments were performed in triplicate. Finally, 450 nm absorbance was measured by a microplate reader. The absorption values were reduced by the blank and normalized to the control wells. Graphs were plotted as the drug concentration versus the percentage of viable cells.

## Supplementary information

A Digital Microfluidic System with 3D Microstructures for Single-Cell Culture

with fences on chip

without fences on chip

Microfluidics: Use of 3D microstructure enables single-cell culture

Supplementary figure
