# Supplementary material for: A digital microfluidic system with 3D microstructures for single-cell culture
Source: Microsyst Nanoeng. 2020 Jan 27;6:6. doi: 10.1038/s41378-019-0109-7 (PMC8433300; doi:10.1038/s41378-019-0109-7)
Supplement: Supplementary file 1 — A Digital Microfluidic System with 3D Microstructures for Single-Cell Culture [file 41378_2019_109_MOESM1_ESM.docx]

**Electronic Supplementary Information**

**A Digital Microfluidic System with 3D Microstructures for Single-Cell Culture**

Jiao Zhai, ^a^ Haoran Li, ^a, b^ Ada Hang-Heng Wong, ^c^ Cheng Dong, ^a^ Shuhong Yi, ^d^ Yanwei Jia, ^a, b, c^ * Pui-In Mak, ^a, b^ Chuxia Deng, ^c^ and Rui Martins. ^a, b, e^

^a^ State-Key Laboratory of Analog and Mixed-Signal VLSI, Institute of Microelectronics, University of Macau, Macao SAR, China.

^b^ Faculty of Science and Technology-ECE, University of Macau, Macau SAR, China

^c^ Cancer Center, Faculty of Health Sciences, University of Macau, Macau, China

^d^ Liver Transplantation Center, the Third Affiliated Hospital, Sun Yat-sen University, Guangzhou 510000, China

^e^ on leave from Instituto Superior Técnico, Universidade de Lisboa, Portugal

* Correspondence: Yanwei Jia (yanweijia@um.edu.mo)


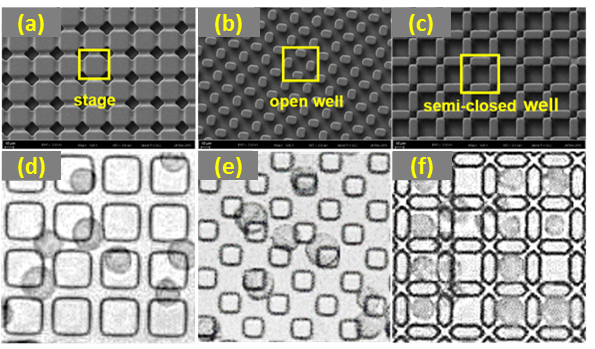


Figure S1. SEM results and the related cell trapping results of the microstructure arrays. (a-c) The SEM results of microstructure arrays. (a) stage array, (b) post array, (c) wall array. (d-f) The corresponding results of trapping MDA-MB-231 cells under different structures (a–c) after 24 h.

Fig. S1 (a-c) show the SEM results of the designed stage array (Fig. S1a), post array (Fig. S1b) and wall array (Fig. S1c). In the stage array design (Fig. S1a), the yellow frame represents one 20 μm stage with a gap between stages of 10 μm. As can be seen from the MDA-MB-231cell distribution after 24 h culturing (Fig. S1d), only a few cells stayed on the stage and most of the cells showed irregular distribution. Some were inclined to stay in the gap between two stages. Others stepped over the stage and the gap. In other words, this structure did not contribute to trapping single cells. This may be due to the small gap between the stages, causing a slightly curved interface at the gap (between two stages), not stable enough to trap single cells. In the post array design (Fig. S1b), the stage was 10 μm with a gap of 20 μm in between. We hypothesized the virtual wells would be big enough for cell accommodation. Noticing the open space of each virtual well spot, this virtual well was named open well, as represented in the yellow frame. We expected the cells would stay in because the oil could freely flow into and out of the structures, promoting the formation of the virtual wells. Under the effect of gravity, cells will stay at the lower interface of the open well. However, the result in Fig. S1e shows that the cells preferred to stay between two adjacent posts instead of the open well. This may be attributed to the oil flow that was strong enough to push the cells through the posts to where the cells got stuck. In the wall array design, as shown in Fig. S1c, the 3D microstructures were patterned as walls with a width of 10 μm, a length of 20 μm and a height of 10 μm. There was a small gap of 5 μm between the ends of each wall, forming a semi-closed well between the walls, as shown in the yellow frame in Fig. S1c. The single cell trapping results are shown in Fig. S1f. As can be seen, cells were perfectly isolated from each other and stored in each semi-closed well.





Figure S2. The normalized fluorescent results after measured on a certain electrode before and after transposing 10 and 100 mg/ml eGFP droplet for 10 times, 50 times or till the droplet stopped moving under the conditions of 0.1% F127 and Silicone oil(1cst) & 0.1% F127.


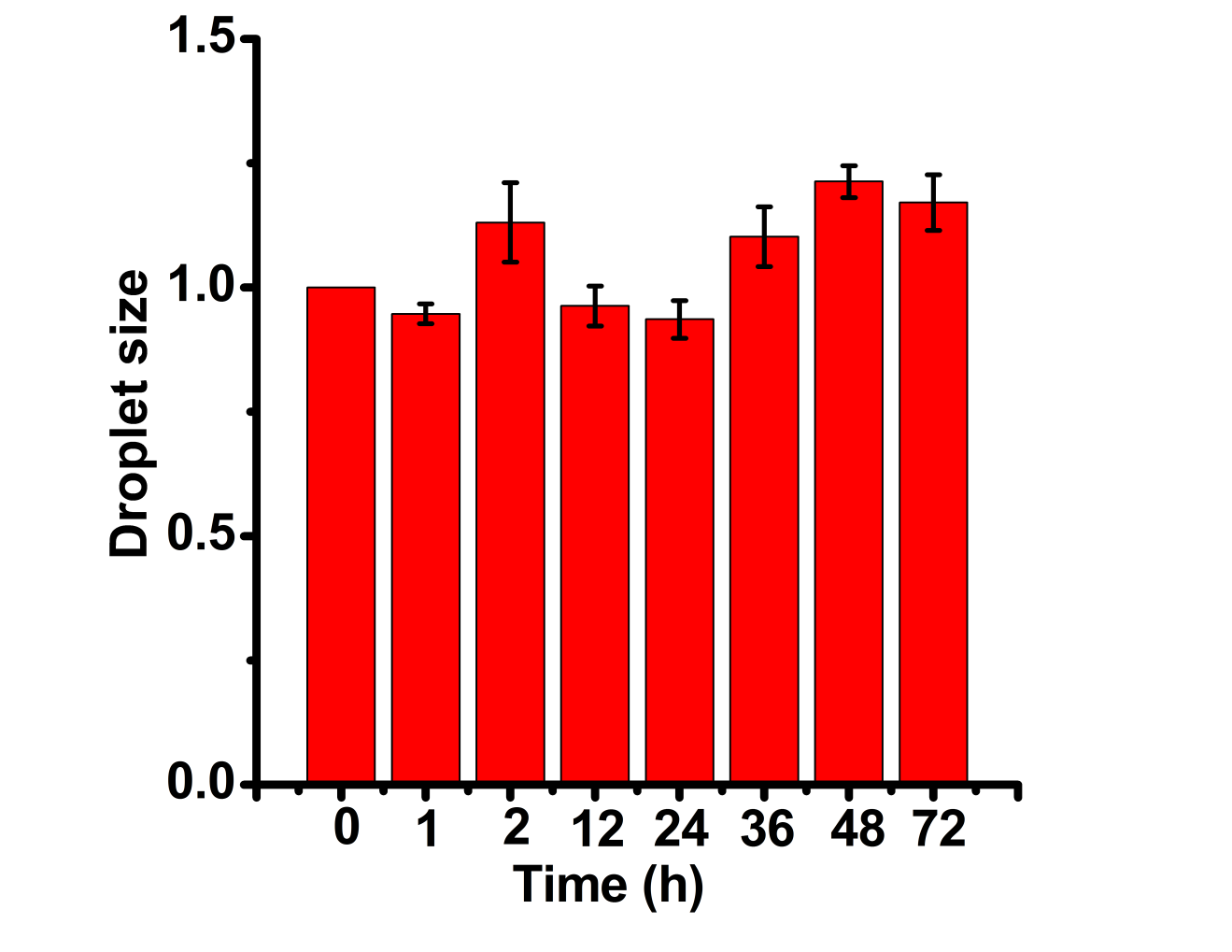


Figure S3. Image J analysis results of the droplet size under the optimized cell culture condition on DMF chip for 72 h.


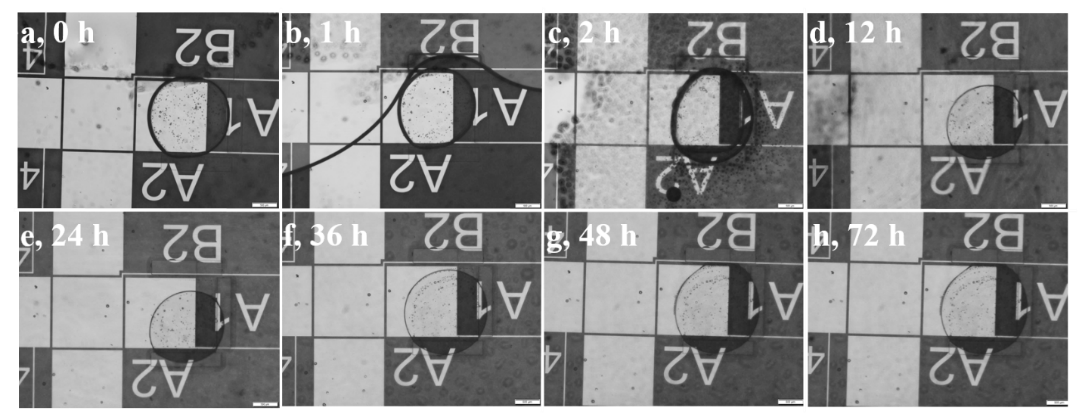


Figure S4. Time dynamics research of the cell suspension under the optimized cell culture condition on DMF chip for 72 h.


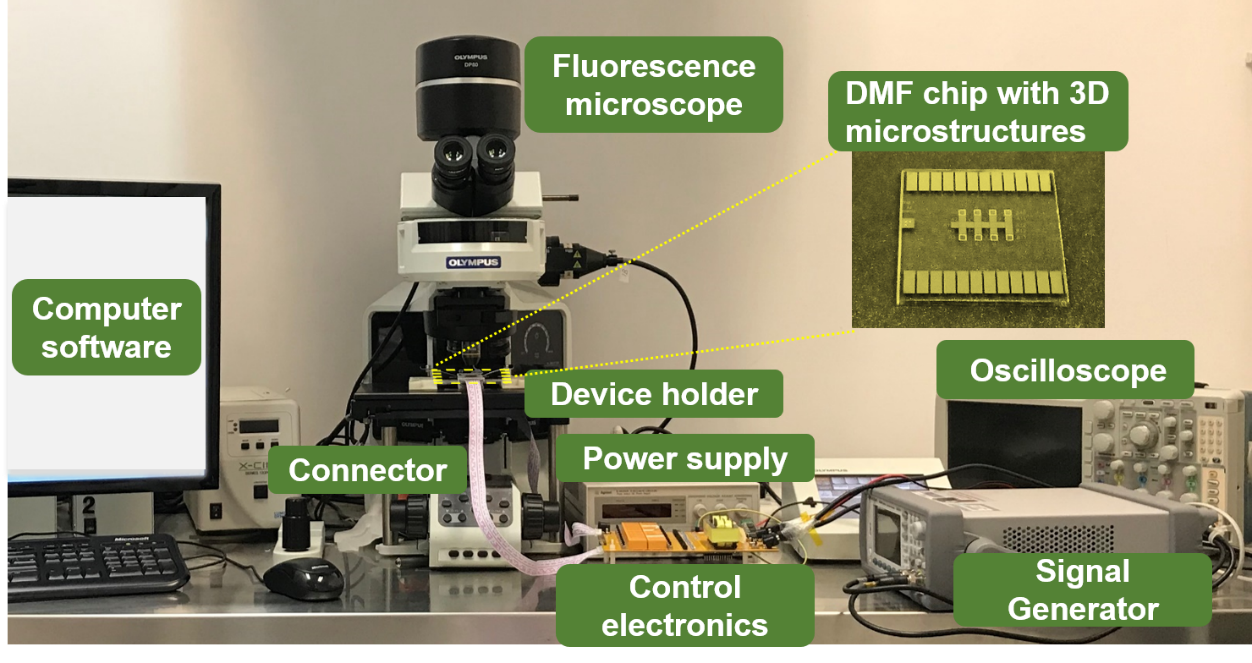


Figure S5. System setup. The DMF system contained four parts: a DMF chip, control electronics (power supply, signal generator, transformer, FPGA, physical relays and connectors), a self-written control software and a fluorescence microscope.


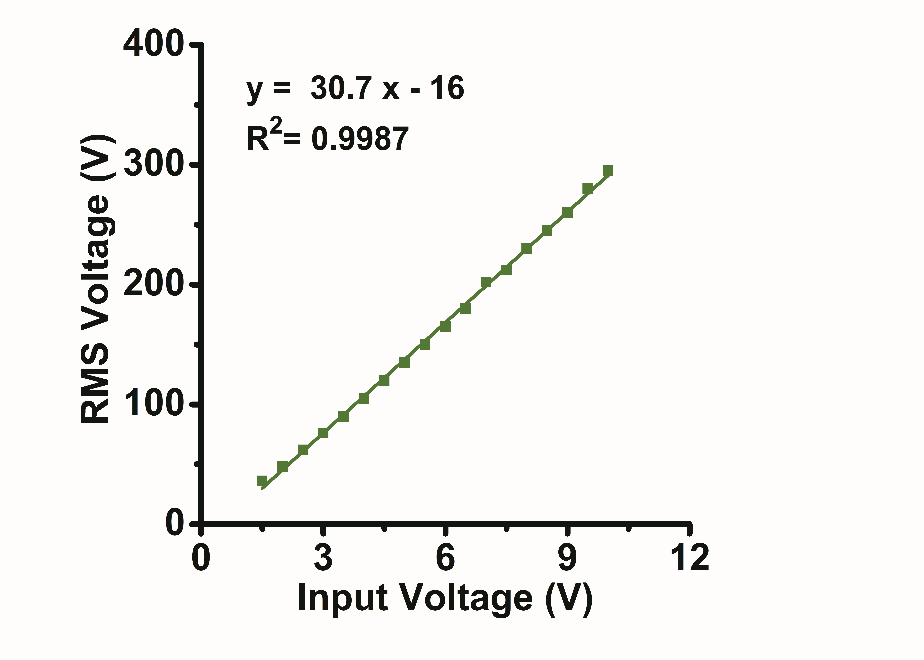


Figure S6. The relationship between input voltage and output voltage after amplification.


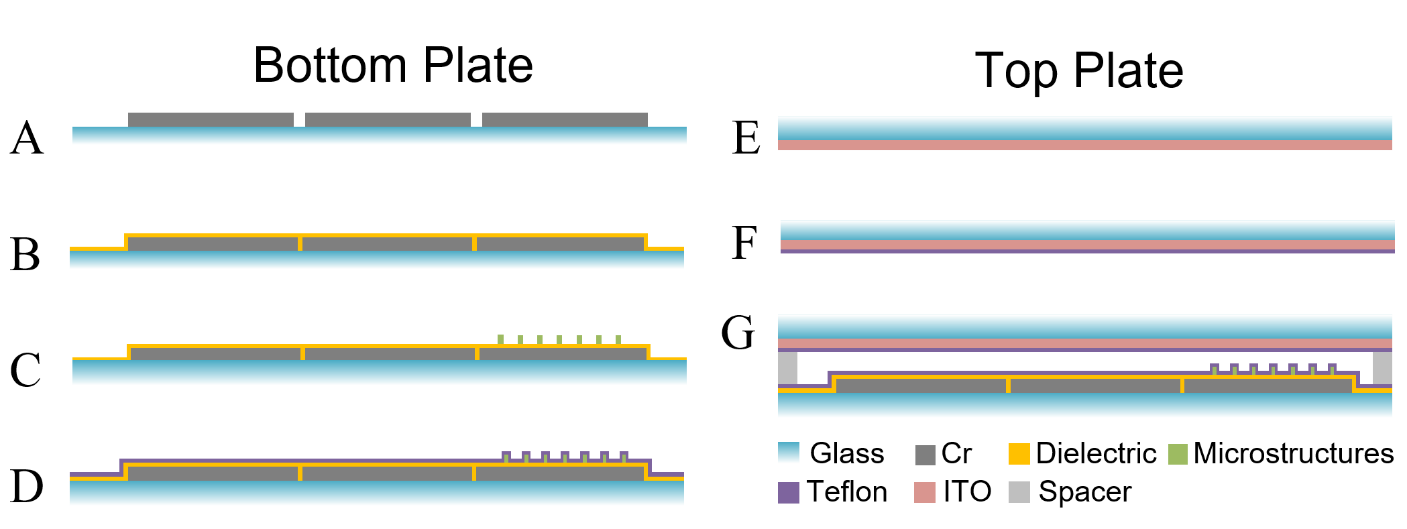


Figure S7. The assembling of DMF chip with microstructure arrays. (A-D) The process for bottom plate construction. (E-F) The construction of top plate (ITO glass coated with teflon). (G) Bottom plate and top plate was assembled with spacer to form a DMF chip.
