# Supplementary material for: A digital microfluidic system with 3D microstructures for single-cell culture
Source: Microsyst Nanoeng. 2020 Jan 27;6:6. doi: 10.1038/s41378-019-0109-7 (PMC8433300; doi:10.1038/s41378-019-0109-7)
Supplement: Supplementary file 4 — Microfluidics: Use of 3D microstructure enables single-cell culture [file 41378_2019_109_MOESM4_ESM.docx]

# *Microsystems & Nanoengineering*

Microfluidics: Use of 3D microstructure enables single-cell culture

A novel microfluidic device enables researchers to isolate and culture single cells for use in drug testing and other experiments. To date, the high voltages required to capture cells on electrodes of a digital microfluidic (DMF) device have led to a reduced lifespan for the device and potentially damaged the cells. A team led by Yanwei Jia of the University of Macau overcame this by incorporating 3D microstructures into a DMF chip. This constrains the shape of cell culture droplets to isolate and capture single cells. With the use of low evaporation temperature oil and a surfactant, the system enabled them to use one quarter of the voltage of other designs for droplet transportation. The ability to isolate and culture individual cells will be of great value in addressing biological questions at the single-cell level.

Related article manuscript number: MICRONANO-00787R

Article title: A Digital Microfluidic System with 3D Microstructures for Single-Cell Culture

Corresponding author and affiliation/s: Yanwei Jia, University of Macau, Macau, Macau

**About your Editorial Summary — please read**

**Before approving this Editorial Summary, please carefully check that (1) the summary text lists the correct author(s) and (2) the spelling and order of all author names and affiliations are correct.**

This **Editorial Summary** is based on your manuscript that was recently accepted for publication in *Microsystems & Nanoengineering*. It provides a non-specialist audience with a synopsis of your key research outcomes and conclusions. This value-added service provided by Springer Nature is designed to raise interest in your research across the broader community.

Springer Nature will publish the summary on the journal’s website, and it will be freely available under a under the CC BY licence (Creative Commons Attribution v4.0 International Licence) (see the journal website for details). We encourage you to re-use the summary to bring attention to your research; for example, you can host it on your own website and share it via social-networking platforms. Please attribute the summary to *Microsystems & Nanoengineering* and your article (e.g. by providing a link to your article) and do not make derivatives.

Please note that to maximise the usefulness of these summaries they must follow several stringent guidelines:
-- Spelling, punctuation and style are set according to *Nature* editorial guidelines. As this summary is aimed at non-expert readers, some concepts and technical terms will be simplified.
-- Total length must be no more than 135 words. It is likely that not all points in the paper will be covered.
-- The first sentence must be no more than 280 characters, including spaces, to allow use on microblogging sites.
-- The headline must consist of a brief generic subject identifier followed by a short description. No more than 10 words in total.

Please contact the editorial office ([mems_nano@mail.ie.ac.cn](mailto:mems_nano@mail.ie.ac.cn)) immediately with corrections should you find any factual errors in this Editorial Summary.
