# Supplementary figures and images for: A digital microfluidic system with 3D microstructures for single-cell culture
Source: Microsyst Nanoeng. 2020 Jan 27;6:6. doi: 10.1038/s41378-019-0109-7 (PMC8433300; doi:10.1038/s41378-019-0109-7)

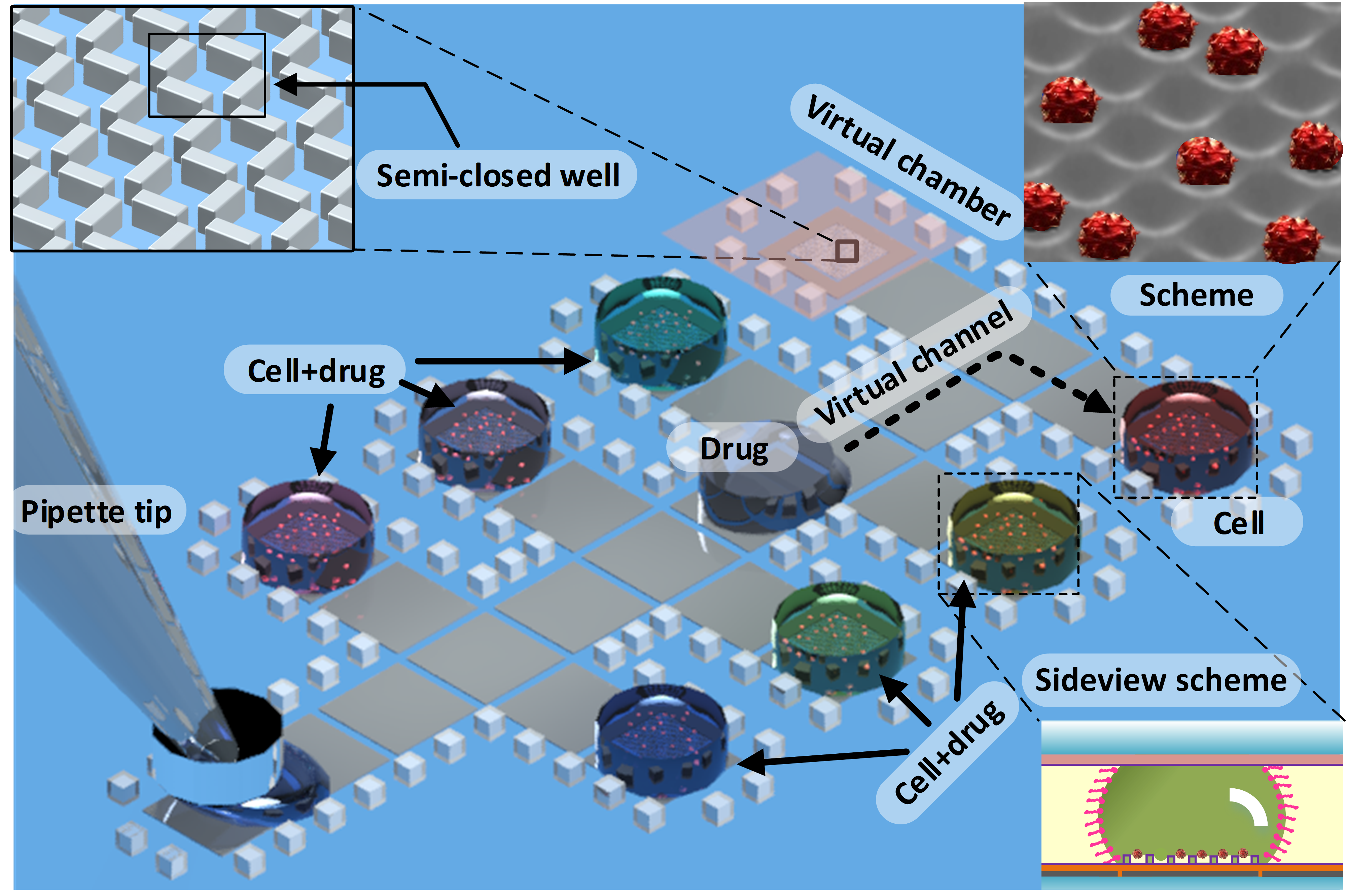

Supplement: Supplementary file 5 — Supplementary figure [file 41378_2019_109_MOESM5_ESM.tif]
